# Reduced cystatin C-estimated GFR and increased creatinine-estimated GFR in comparison with iohexol-estimated GFR in a hyperthyroid patient: A case report

**DOI:** 10.1186/1752-1947-2-66

**Published:** 2008-02-28

**Authors:** Malgorzata Karawajczyk, Mia Ramklint, Anders Larsson

**Affiliations:** 1 Department of Medical Sciences, Clinical Chemistry, Uppsala University Hospital, Uppsala, Sweden; 2 Department of Neuroscience, Psychiatry, Uppsala University Hospital, Uppsala, Sweden

## Abstract

**Introduction:**

Estimation of the glomerular filtration rate (GFR) is essential for the evaluation of patients with kidney disease, and for treating patients with drugs that are eliminated from the circulation by the kidneys. Cystatin C has been shown to be superior to creatinine for estimating GFR in several studies. However, studies showing that thyroid function has an impact on cystatin C have not addressed the question of whether the changes in cystatin C levels are due to changes in GFR or in cystatin C synthesis.

**Case presentation:**

We report an account of a hyperthyroid patient with a discrepancy between the GFR estimates from cystatin C and creatinine. The cystatin C concentration (1.36 mg/L) was higher and gave an estimated GFR which was lower (51 mL/min/1.73 m^2^), while the creatinine concentration was lower (36 μmol/L) and gave a corresponding creatinine-estimated GFR that was higher (145 mL/min/1.73 m^2^) than the iohexol-estimated GFR (121 mL/min/1.73 m^2^) during the hyperthyroid period. After thyroidectomy, the creatinine concentration was 36 μmol/L and creatinine-estimated GFR was calculated as 73 mL/min/1.73 m^2^, while the cystatin C concentration and cystatin C-calculated GFR was 0.78 mg/L and 114 mL/min/1.73 m^2^, respectively.

**Conclusion:**

In contrast to creatinine, cystatin C levels rose in the hyperthyroid state as compared to the euthyroid state. The cystatin C-estimated GFR was reduced compared to the iohexol-estimated GFR. This patient case shows that the hyperthyroid-associated changes in cystatin C levels are not due to changes in GFR. Thyroid function should thus be considered when both cystatin C and creatinine are used as markers of kidney function.

## Introduction

Glomerular filtration rate (GFR) is generally accepted as the best overall indicator of renal function and is therefore an important marker for renal disease. Reduced GFR influences the clearance of many pharmaceuticals used today. In the last decades, serum or plasma creatinine has become the most commonly used marker for estimating GFR in clinical practice [1,2]. Despite common use, creatinine has serious limitations as a marker for renal function. GFR is often calculated from plasma creatinine using the Cockcroft-Gault [3] or Modification of Diet in Renal Disease (MDRD) study equations [4]. It is recommended that laboratories should report estimated GFR instead of only reporting the concentration of the analyte [5]. Creatinine is also influenced by factors such as age, gender, muscle mass, thyroid function and physical activity [6]. Cystatin C is a cysteine protease inhibitor with a molecular mass of 13 kDa [7]. A recent meta-analysis indicated that cystatin C is superior to plasma creatinine as a marker for estimating renal function [8]. Serum cystatin C concentration is reported to be unaffected by muscle mass, age, inflammation, fever or exogenous agents [9,10]. However, there are reports that thyroid function has an impact on both cystatin C and creatinine levels [11]. Serum creatinine values have been shown to be higher in hypothyroidism, and lower in hyperthyroidism, as compared to the euthyroid state. For cystatin C levels, the contrary was reported in these patient groups [12]. These cystatin C alterations are probably due to changes in the synthesis of cystatin C, but could also be due to changes in clearance as suggested for creatinine. There are no reports on the correlation between cystatin C alterations in hyperthyroid patients and GFR measured with an exogenous marker. Thus, in a hyperthyroid patient, we compared cystatin C with iohexol clearance.

## Case presentation

### Case description

A Caucasian female (age 25), smoker, was admitted to the emergency unit with symptoms of tachycardia and anxiety. Thyroid tests were ordered on suspicion of hyperthyroidism. The test results showed values characteristic of severe hyperthyroidism: TSH was below the detection limit of the assay (<0.001 mU/L), 40 pmol/L free T4 (ref. interval 10–18 pmol/L) and 6.4 nmol T3/L (ref. interval 1.2–2.8 nmol/L). Antibody levels against thyroid peroxidase (anti-TPO) and TSH receptor were increased as follows: 29 kIU anti-TPO/L (ref. interval <16) and 11 anti-TSH receptor U/L (ref. interval <0.6 U/L). The patient was diagnosed with Graves's disease showing disseminated thyroid enlargement. Initial treatment included a β-receptor blocker, propranolol and an inhibitor of thyroxin synthesis called timazol. Later, a thyroidectomy was performed.

Plasma levels of creatinine and cystatin C were analyzed as markers of kidney function. When the patient was in the hyperthyroid state, plasma creatinine was 36 μmol/L (ref. interval 50–90 μmol/L) and cystatin C was 1.36 mg/L. Calculated glomerular filtration rate (GFR) from creatinine was 145 mL/min/1.73 m^2 ^according to the bias-corrected MDRD equation [13] and 51 mL/min/1.73 m^2 ^as calculated from cystatin C [14]. A subsequent iohexol clearance was performed two days later due to the disproportion between the two GFR estimations. The GFR determined by iohexol clearance during the hyperthyroid period was 121 mL/min/1.73 m^2^.

Six months later (after surgery), the patient was clinically euthyroid with a TSH value of 3.69 mIE/L and 15 pmol free T4/L. Creatinine and cystatin C levels were 65 μmol/L and 0.78 mg/L, respectively. GFR-calculated from the creatinine concentration was 73 mL/min/1.73 m^2 ^compared to 114 mL/min/1.73 m^2 ^using the cystatin C concentration.

GFR was determined by measuring the plasma clearance of iohexol, an x-ray contrast agent that is a reliable marker for GFR. The patient was given 5 mL iohexol solution (Omnipaque, Nycomed Amersham) intravenously in an antecubital vein. The GFR was calculated from the iohexol concentration after the injection. Serum iohexol levels were determined by high-pressure liquid chromatography. The total analytical imprecision of the method was 2.5% using a control sample with an assigned value of 33.5 mg/L and 2.4% for a control sample with an assigned value of 65.7 mg/L.

Plasma creatinine measurements were performed by means of the modified kinetic Jaffe reaction on an Architect ci8200 analyzer (Abbott Laboratories, Abbott Park, IL, USA) and reported using S.I. units (μmol/L). The total analytical imprecision of the creatinine method was 3.2% both at 94 and 337 μmol/L.

Cystatin C immunoparticles (code 1014), assay buffer (code 1007), calibrator (code 1012) and control set (code 1019) were all obtained from Gentian (Moss, Norway). The immunoparticles are coated with purified chicken antibodies to cystatin C on uniform polystyrene particles. Plasma cystatin C measurements on the Architect ci8200 was performed using the following instrument settings: primary wavelength 548 nm, secondary wavelength 700 nm, sample blank position 18, and spline calibration method. 220 μL reagent 1 and 3 μL sample were mixed with 45 μL reagent 2. The total analytical imprecision of the cystatin C method was 1.7% at 0.77 mg/L, 1.1% at 1.25 mg/L and 1.4% at 5.45 mg/L.

TSH, free T4 and T3 were also analyzed using the Architect ci8200. The measuring range of the TSH method was 0.001–100 mU/L. The total analytical imprecision of the TSH method was 2.4% at 0.52 mU/L and 2.4% at 5.07 mU/L. The total analytical imprecision was 7.7% at 0.81 nmol/L and 2.53% at 4.06 nmol/L for the T3 method, and 5.2% at 11.5 pmol/L and 4.4% at 16.8 pmol/L for the free T4 method.

Anti-TPO was analyzed on a Liaison instrument (DiaSorin Laboratories, Saluggia, Italy). The total analytical imprecision of the anti-TPO method was 5.8% at 17.7 kU/L and 8.1% at 161.8 kU/L. Anti-TSH receptor antibodies were analyzed with an ELISA kit from RSR Ltd. (Cardiff, United Kingdom).

## Discussion

Hypo- and hyperthyroid diseases occur frequently. Hence, it is important to be aware of erroneous results due to these diseases. The cystatin C-estimated GFR (51 mL/min/1.73 m^2^) during the hyperthyroid period was lower than the corresponding iohexol-estimated GFR (121 mL/min/1.73 m^2^). On the other hand, the cystatin C-estimated GFR postoperatively (114 mL/min/1.73 m^2^) was in close agreement with the iohexol-estimated GFR during the hyperthyroid period. This indicates that the increase in cystatin C and subsequent decrease in cystatin C-estimated GFR is not due to a change in glomerular clearance, but rather due to an increased synthesis related to the hyperthyroid state. Cystatin C is considered to be produced in all nucleated cells at a constant rate. No iohexol clearance was performed in the euthyroid state. The iohexol clearance performed during the hyperthyroid state was part of the patient's treatment to evaluate whether the patient had a decreased GFR or not. During the euthyroid state, the cystatin C-estimated GFR was 114 mL/min/1.73 m^2 ^and the creatinine-estimated GFR was 73 mL/min/1.73 m^2^. According to previous reports, the MDRD equation should only be used to report numeric results below 60 mL/min/1.73 m^2 ^[15]. Thus, both GFR estimates in the euthyroid period were considered normal, and it was therefore not considered ethical to perform an additional iohexol clearance during the euthyroid period.

The creatinine values (36 μmol/L) in this patient were reduced during the hyperthyroid period with an ensuing catabolic state resulting in an overestimation of the creatinine-calculated GFR (145 mL/min/1.73 m^2^). Thus, both cystatin C and creatinine gave erroneous GFRs in comparison to iohexol clearance, which was used as the reference method.

## Conclusion

The alteration in cystatin C level is not due to a change in GFR in connection with hyperthyroidism. Accordingly, altered thyroid function should be considered both when creatinine and cystatin C is used for determining kidney function.

## Competing interests

The author(s) declare that they have no competing interests.

## Authors' contributions

MR was responsible for patient care and data collection. MK and AL conceived the research question. MK and AL wrote the first draft. Subsequent revisions were carried out with input from all authors. The final version was approved by all authors.

## Consent

Written consent was obtained from the patient for publication of the study.
